# Effects of *Gingko biloba* and Milk Thistle Extracts on Biomarkers of Antioxidants Status and Liver Function in Healthy Dogs

**DOI:** 10.3390/vetsci12080763

**Published:** 2025-08-16

**Authors:** Rachakris Lertpatarakomol, Tassanee Trairatapiwan, Auraiwan Klaengkaew, Jamlong Mitchaothai, Achara Lukkananukool

**Affiliations:** 1 Faculty of Veterinary Medicine, Mahanakorn University of Technology (MUT), Bangkok 10530, Thailand; rachakris@mut.ac.th (R.L.); auraiwan@mut.ac.th (A.K.); 2 Office of Administrative Interdisciplinary Program on Agricultural Technology, School of Agricultural Technology, King Mongkut’s Institute of Technology Ladkrabang (KMITL), Bangkok 10520, Thailand; jamlong.mi@kmitl.ac.th; 3 Department of Animal Production Technology and Fisheries, School of Agricultural Technology, King Mongkut’s Institute of Technology Ladkrabang (KMITL), Bangkok 10520, Thailand; achara.lu@kmitl.ac.th

**Keywords:** *Gingko biloba*, milk thistle, antioxidants, liver function, healthy dog

## Abstract

This study investigated the effects of *Ginkgo biloba* and milk thistle seed extract (GB/MT) supplementation on antioxidant status, liver function, and general health in healthy dogs. Six French Bulldogs were enrolled in a crossover design and fed commercial diets supplemented with GB/MT at either 1 or 2 g/day for 21 days. Blood samples were collected at baseline (day 0), during supplementation (days 14 and 21), and after withdrawal (day 28) to assess serum superoxide dismutase (SOD), glutathione (GSH/GSSG), liver enzymes (ALT, AST, ALP), albumin, total protein, blood urea nitrogen, creatinine, triglycerides, and cholesterol. Results showed that GB/MT supplementation had no significant effect on antioxidant markers (SOD and GSH/GSSG) at either dose. However, both supplementation levels led to significant decreases in ALT and increases in serum albumin levels after 21 days. No significant differences were observed in other liver function or general health parameters. These findings suggest that GB/MT supplementation does not enhance antioxidant enzyme activity under non-stressed conditions but may support liver health by reducing liver enzyme levels and enhancing protein synthesis. The 1 g/day dose was found to be as effective as the higher dose, suggesting a cost-effective approach for maintaining liver function in dogs through dietary botanical supplementation.

## Simple Summary

Pet owners are increasingly interested in natural supplements to maintain their dogs’ health. This study looked at whether two plant-based extracts, *Ginkgo biloba* and milk thistle, can help improve liver function and antioxidant levels in healthy dogs. Six French Bulldogs were given either 1 or 2 g/day of the combined extracts for 21 days. Blood tests were performed before, during, and after the supplementation period. The study found that the supplements did not change antioxidant levels, but they did reduce a liver enzyme (ALT) and increased a protein called albumin, which is made by the liver. These changes suggest that the supplements may support liver health, even in healthy dogs. Importantly, both the low and high doses had similar effects, meaning that the lower dose may be just as beneficial and more cost-effective. This research adds to the growing evidence that certain natural supplements can play a role in supporting canine well-being.

## 1. Introduction

The use of botanical extracts in companion animal nutrition has garnered increasing scientific interest due to their potential roles in promoting health and mitigating disease through natural mechanisms. Among these, *Ginkgo biloba* (GB) and milk thistle (*Silybum marianum*, MT) are two widely recognized herbal agents known for their antioxidant and hepatoprotective properties. *Ginkgo biloba* contains phenolic compounds, primarily flavonoids and terpenoids, which have been demonstrated to enhance antioxidant defenses, improve microcirculation, and modulate hepatic enzyme activity [1,2,3]. It has also been associated with improved cognitive performance and neuroprotection in both humans and animals, suggesting broader systemic benefits. Similarly, MT, particularly its active phenolic compound silymarin, is noted for stabilizing hepatocyte membranes, promoting protein synthesis, scavenging free radicals, and inhibiting lipid peroxidation [4,5,6,7,8]. These phytochemicals may work synergistically to support cellular resilience and mitigate oxidative stress induced by metabolic and environmental challenges.

*Ginkgo biloba* extract has demonstrated a generally safe profile in dogs when used at appropriate doses. A clinical study by Reichling et al. [9] reported significant improvement in cognitive symptoms in elderly dogs receiving 40 mg/10 kg body weight (BW)/day for 8 weeks, with no serious adverse effects. The European Food Safety Authority (EFSA) also concluded GB is safe for dogs at up to 3.3 mg/kg of complete feed, provided the ginkgolic acid content remains below 1 mg/kg. However, potential side effects include gastrointestinal upset, allergic reactions, and bleeding risk due to platelet-activating factor (PAF) inhibition [10]. *Ginkgo biloba* should be avoided in dogs with seizure history, bleeding disorders, or those on nonsteroidal anti-inflammatory drugs (NSAIDs) or anticoagulants, and is not recommended during pregnancy or lactation [11].

In dogs, MT is considered safe and beneficial for canine liver support when used under veterinary guidance, though further clinical research is warranted to refine dosing and long-term safety profiles. The typical therapeutic dose of MT (silymarin) in dogs is 5–20 mg/kg BW per day, often divided into 1–2 doses. For enhanced formulations like silybin–phosphatidylcholine complexes, lower doses (2–10 mg/kg/day) may be effective due to better absorption. Treatment duration varies by condition but generally ranges from 2 to 8 weeks [12,13]. The supplement is well-tolerated at these doses under veterinary supervision. Studies confirm its good tolerability with a high oral safety margin, LD_50_ values exceeding 10 g/kg BW, and no significant toxicity has been reported at therapeutic doses [12,14]. Adverse effects are rare but may include mild gastrointestinal upset, such as diarrhea or appetite changes. Silymarin has been shown to inhibit certain cytochrome P450 enzymes, such as CYP3A4 and CYP2C9, which are hepatic enzymes responsible for approximately 90% of drug metabolism. However, at the plasma concentrations typically achieved through oral administration, clinically relevant drug interactions are considered unlikely [12]. Furthermore, MT is contraindicated in pregnant or lactating dogs, puppies, dogs with known allergies to plants in the Asteraceae family, and those receiving cisapride due to potential cardiac risks [6,12,13].

Moreover, it has been reported that polyphenols generally exhibit a high level of safety. However, polyphenols extracted from green tea can be toxic at doses ≥ 200 mg/kg/day when administered in a fasted state, leading to gastrointestinal, hepatic, and renal damage, whereas co-administration with food significantly reduces these adverse effects [15]. In contrast, mixed polyphenol extracts, such as grape and blueberry blends, have demonstrated long-term safety at doses up to 40 mg/kg/day. Therefore, the use of polyphenol supplements in dogs should take into account the specific compound, dosage, and feeding conditions to ensure safe application [16].

Although these effects have been well-documented in rodent models [17,18], livestock, and companion animals [19,20,21], and even in clinical settings involving hepatic dysfunction in veterinary medicine [22,23,24], their implications for healthy canines remain underexplored. Limited research has addressed how such supplements might influence physiological markers in non-diseased dogs, particularly breeds with predispositions to hepatic or metabolic disorders. Additionally, the bioavailability and efficacy of these herbal constituents may vary by species, age, diet, and formulation, necessitating targeted studies to confirm their benefits in canine populations.

Considering the rising demand for functional pet foods and natural health supplements, evaluating the physiological impacts of such extracts in dogs is both timely and necessary. Pet owners increasingly seek natural alternatives to conventional medications, emphasizing the importance of scientific validation for these ingredients.

This study aimed to investigate the effects of dietary supplementation with the combination of GB and MT seed extracts (GB/MT) on biomarkers related to antioxidant status, liver function, renal function, and general health in dogs. Specifically, this study assessed changes in serum superoxide dismutase (SOD), glutathione (GSSG/GSH), and liver enzymes, including alanine transaminase (ALT), alkaline phosphatase (ALP), and aspartate transaminase (AST), as well as biochemical markers such as albumin, total protein, blood urea nitrogen (BUN), creatinine, triglycerides, and cholesterol. French Bulldogs enrolled in the experiment were fed commercial diets supplemented with varying doses of the plant extracts over a 21-day period. The results provide insight into the nutraceutical potential of these botanicals in canine health maintenance and may inform the development of evidence-based dietary interventions aimed at supporting organ function and systemic balance in dogs.

## 2. Materials and Methods

### 2.1. Animal Cares

The present experiment was reviewed and approved by the Institutional Animal Care and Use Committee of Mahanakorn University of Technology (ACUC-MUT-2024/010).

### 2.2. Animals, Treatments, and Experimental Design

The experiment was conducted by enrolling six healthy French Bulldogs, aged 2–3 years, average BW 14 ± 2 kg, including two intact males and four intact females. The experimental treatments were divided into 2 groups of 6 replicates in each, as a change-over design, consisting of group 1, dogs fed commercial diet supplemented with GB/MT (commercial product) at 1 g/day for 21 days; and group 2, dogs fed commercial diet supplemented with GB/MT at 2 g/day for 21 days. Each kilogram of GB/MT mixture contains a total of 200 mg of polyphenols.

This study was conducted at the research facility of the Faculty of Veterinary Medicine, Mahanakorn University of Technology. The criteria for selecting clinically healthy dogs for inclusion in the study were based on BW, normal behavior, and general physical and physiological assessments conducted by a veterinarian. Following this initial screening, all dogs underwent liver ultrasound examinations, including evaluation of the liver surface, parenchyma, and biliary tract to further assess hepatic function prior to enrollment. The dogs were individually housed in cages measuring 1.3 × 1.2 × 0.9 m within a 24 m^2^ climate-controlled room.

Blood samples were collected on day 0 (before supplementation) to assess antioxidant markers (SOD and GSSG/GSH), liver function (ALT, AST, ALP, and albumin), renal function (BUN and creatinine), and blood biochemistry (cholesterol and triglycerides). Following this, GB/MT supplementation was administered according to group allocation.

The commercial diet, formulated for adult large breed dogs, contains not less than 25% protein, not less than 13% fat, not more than 4% fiber, not more than 10% moisture, and provides 375 kilocalories of metabolizable energy (ME) per 100 g. The amount of food given to each dog was calculated from the formula, maintenance energy (ME) requirements (calories per day) = 132 × BW (kg)^0.75^, and given twice a day at 8:00 and 16:00 with fresh water for the dog to drink at all times. The GB/MT supplement was administered once daily by thoroughly mixing the assigned dose (1 g or 2 g) into the morning meal prior to feeding. This ensured complete consumption of the supplement along with the diet. The dogs were individually fed to allow for accurate intake monitoring and to ensure consistent dosage across the study period. The dogs had daily access to 30 min of free activity outside the cage after the morning meal. The washout period between phase 1 and phase 2 of the crossover design comprised a total of 14 days. This included a 7-day withdrawal period at the end of phase 1, during which supplementation was discontinued, followed by an additional 7-day rest period before commencing the next treatment phase.

Feed intake (FI) was recorded daily. The dogs’ health status was monitored every morning and evening. Blood samples (5 mL each) were collected on days 0, 14, 21, and 28 (i.e., seven days after withdrawal of supplementation) to evaluate antioxidant status, liver and renal function, and blood biochemical parameters

### 2.3. Determinations of Liver and Blood Parameters

In this study, SOD activity was measured using the SOD Assay Kit (Catalog No. 19160-1KT-F; Sigma-Aldrich, Thermo Fisher Scientific Inc., Waltham, MA, USA), while glutathione levels (GSH/GSSG) were determined using the Glutathione Assay Kit (Catalog No. MAK440-1KT; Sigma-Aldrich, Thermo Fisher Scientific Inc., Waltham, MA, USA). Serum biochemical parameters were analyzed using a Thermo Konelab 20i Chemistry Analyzer (Thermo Fisher Scientific Inc., Waltham, MA, USA).

### 2.4. Statistical Analysis

Data collected at different time points (day 0, 14, 21, and after a 7-day withdrawal period on day 28) were analyzed using repeated measures ANOVA. The effects of supplementation levels (1 g/day and 2 g/day) were analyzed using an independent t-test. All statistical analyses were performed using SPSS^®^ software version 23 (SPSS Inc., Chicago, IL, USA).

## 3. Results

### 3.1. Complete Blood Count and Liver Function Status of Dogs Before Starting the Experiment

Before starting the experiment, a complete blood count and liver function tests were conducted, including an ultrasound to examine the liver surface, parenchyma, and biliary tract. All dogs used in the experiment had normal blood count values and showed no signs of liver disease.

### 3.2. Effects of Supplementation of GB and MT Extract in Diets on FI and BW in Dogs

The effects of supplementing GB/MT in diets on FI and BW in dogs are summarized in Table 1. The results showed no significant differences in FI across the weeks of the experiment (*p* > 0.05). Similarly, BW of dogs at both the beginning and end of the experiment showed no significat difference between the groups (*p* > 0.05).

### 3.3. Effects of Supplementation of GB and MT Extract at Different Periods in Diets on Antioxidant Indicators in Dogs

The effects of supplementing GB/MT at different time points in diets on antioxidant indicators in dogs are summarized in Table 2. In this study, serum SOD levels were used as the primary antioxidant indicator. The results showed no significant differences (*p* > 0.05) at any time point: before supplementation, after 14 and 21 days of supplementation, and after 7 days of ending between the two groups receiving 1 and 2 g/d of the supplement.

### 3.4. Effects of Dietary Supplementation of GB and MT Extract on Liver Function and Health Indicators in Dogs

Table 3 showed the effects of dietary supplementation of GB/MT at 1 g/d on liver function and health indicators in dogs. Supplementing with 1 g/d for 21 days resulted in lower serum ALT values compared to before supplementation (*p* < 0.05). However, ALT values after 14 days of supplementation and 7 days after cessation were not significantly different from baseline (Day 0, *p* > 0.05). Similarly, serum albumin levels were higher after 21 days of supplementation at 1 g/d compared to before supplementation (*p* < 0.05). No significant difference in albumin levels was observed after 14 days of supplementation and 7 days after cessation (*p* > 0.05). No statistically significant differences were found for other liver function indicators (AST, ALP, total protein) or general health indicators (BUN, creatinine, triglycerides, cholesterol) at any time point (*p* > 0.05).

Table 4 showed the effects of dietary supplementation of GB/MT at 2 g/d on liver function and health indicators in dogs. After stopping supplementation at 2 g/d for 7 days, serum ALT values were significantly lower compared to baseline (Day 0, *p* < 0.05). However, ALT values after 14 and 21 days of supplementation showed no significant difference from baseline (*p* > 0.05), although a decreasing trend was observed. Supplementing at 2 g/d for 21 days and 7 days after cessation resulted in higher serum albumin levels compared to baseline (*p* < 0.05), while no significant difference was observed after 14 days of supplementation (*p* > 0.05). No significant differences were observed in other liver function indicators (AST, ALP, total protein) or general health indicators (BUN, creatinine, triglycerides, cholesterol) at any time point (*p* > 0.05). However, triglyceride and cholesterol levels were numerically lower after 14 and 21 days of supplementation at 2 g/d compared to baseline.

### 3.5. Effect of Supplementation Levels of GB and MT Extract in Diets on Liver Function and Health Indicators in Dogs

Table 5 showed the effects of different supplementation levels of GB/MT on liver function and health indicators in dogs. Before supplementation, dogs in the 2 g/d group had significantly higher ALT values compared to those in the 1 g/d group (*p* < 0.05). No significant differences were observed in other liver function indicators (AST, ALP, total protein, albumin) or general health indicators (BUN, creatinine, triglycerides, cholesterol) between the groups (*p* > 0.05). After 14 and 21 days of supplementation, as well as 7 days withdraw period, there were no significant differences in liver function and general health indicators between the 1 and 2 g/d groups (*p* > 0.05).

## 4. Discussion

This study found no significant differences in FI or BW (both initial and final BW) between the 1 and 2 g/d supplementation groups. These findings are consistent with Banin et al. [25], who reported that 14 days of GB supplementation in mice did not affect FI or BW gain compared to a control group. Interestingly, both experimental groups showed lower FI than the amount provided. This could be attributed to the hot weather during the experiment and the dogs’ habitual practice of eating only one meal a day. When the dogs were fed two meals a day (morning at 8:00 a.m. and evening at 4:00 p.m.), leftover food was observed at the evening meal. However, this did not affect the results of the study as the supplementation was only added to the morning meal.

A 14-day washout period was implemented to minimize the risk of carryover effects and to allow adequate time for the clearance of bioactive compounds from the system. No significant differences were observed in baseline (Day 0) values between the two treatment phases, indicating that potential carryover effects were minimal or negligible.

This study found no significant differences in serum SOD levels between the 1 and 2 g/d supplementation groups before supplementation, after 14 and 21 days of supplementation, or 7 days following withdrawal. This is consistent with the findings of Hermenean et al. [5], who reported that 21 days of MT seed extract supplementation at 10 g/kg BW in mice did not alter SOD levels compared to a control group. However, when oxidative stress was induced with carbon tetrachloride injection, the group receiving MT extract showed higher SOD levels than the control. In contrast, Bridi et al. [1] and Ahmed et al. [2] found that administration of GB extract at 100 mg/kg BW for 14 days significantly increased SOD levels in mice. Additionally, Sgorlon et al. [26] reported that supplementation with MT extract at 10 mg/kg BW for 60 days significantly upregulated SOD-2 expression in dogs with hepatic disorders.

Several factors can influence experimental outcomes, particularly SOD enzyme levels, which tend to decrease under conditions of oxidative stress. In such cases, dietary supplementation has been shown to elevate SOD levels [5]. However, in this study, the dogs were maintained under normal care conditions with regular free-range activity and were not exposed to oxidative stress. Consequently, the supplementation did not affect the antioxidant markers. Other contributing factors such as extract preparation methods, supplementation dosage, administration techniques, duration of treatment, as well as the breed and age of the animals may also account for variations in experimental results.

Supplementation with GB/MT at a dose of 1 g/d for 21 days resulted in a statistically significant reduction in serum ALT levels in dogs. ALT values remained lower than baseline (Day 0) after 14 days of supplementation and continued to be reduced even 7 days after the withdrawal. Similarly, supplementation at 2 g/d for both 14 and 21 days also led to a significant decrease in serum ALT levels. Notably, ALT levels remained significantly lower than baseline even 7 days after the withdrawal of the 2 g/d supplementation.

These findings are consistent with those of Sgorlon et al. [26], who reported that dietary supplementation with MT seed extract (silybin at 1.5 mg/kg BW) for 60 days reduced plasma ALT/GPT levels in dogs with liver disease. In another study, Soltanian et al. [27] observed significant reductions in AST and ALP in dogs treated with silybin, indicating its therapeutic effects on clinical and hematological parameters and organ injury. Similarly, Gogulski et al. [17] found that 28 days of silybin extract supplementation reduced serum ALT and AST levels in dogs. These beneficial effects are attributed to the antioxidant, anti-inflammatory, and hepatoprotective properties of MT seed extract [4].

The experiment found that supplementation with 1 g/d of the supplement for 21 days resulted in a statistically significant increase in serum albumin levels in dogs. Albumin levels remained elevated after 14 days of supplementation and 7 days post-withdrawal compared to baseline values. Similarly, supplementation at 2 g/d for 21 days, as well as 7 days after withdrawal, also led to significantly higher serum albumin levels. After 14 days of supplementation, albumin levels were likewise significantly higher than baseline.

These findings are consistent with those of Naik and Panda [28], who reported that GB leaf extract (25 and 50 mg/kg for 10 days) increased albumin and total protein levels in rats with liver dysfunction. Similarly, Gogulski et al. [17] found that silybin extract (2.83 mg/kg) administered for 30 days increased albumin levels in dogs with liver disease. Albumin is a protein synthesized by the liver, and its levels typically decrease in conditions associated with impaired liver function, such as cirrhosis, or in nephrotic syndrome, where albumin is lost through the urine. In this study, supplementation led to elevated albumin levels, suggesting a potential hepatoprotective effect. Therefore, supplementation may support liver function, particularly with respect to protein synthesis.

The experiment measured liver function markers (AST, ALP, and total protein) and found that supplementation at 1 and 2 g/d for 14 and 21 days did not result in statistically significant changes in these values compared to baseline. However, the AST and ALP values showed slight decreases. In contrast, Gogulski et al. [17] found that supplementing with silybin extract at 2.83 mg/kg BW for 30 days resulted in lower AST and ALP values in dogs. Similarly, Martello et al. [29] found that supplementation with silybin extract for 28 days decreased ALT, AST, and ALP values, though total protein levels remained unchanged. Furthermore, in a study conducted by Kocatürk et al. [30], in five healthy dogs treated daily with S-adenosylmethionine (SAMe) + silybin, when exposed to bacterial lipopolysaccharide (LPS) to cause endotoxemia, the increase in liver enzymes associated with the effects of LPS were inhibited at 1–24 h by the treatment with SAMe + silybin.

Among liver enzymes, ALT is more specific to liver function, whereas AST and ALP are also present in other organs, such as the brain, heart, kidneys, and muscles. ALP, on the other hand, is linked to bile duct and bone function. The lack of change in total protein levels may be because protein production occurs in multiple organs, not just the liver. Thus, while the extract supplementation may help restore liver function and increase albumin production, changes in total protein levels may not be immediately evident, as other proteins produced by organs outside the liver remain unchanged [31].

Although the dogs initially exhibited mildly elevated ALT levels above the normal range, most parameters remained within normal limits, indicating no clinical signs of impaired liver function. While the parameters did not exceed clinical thresholds indicative of hepatic dysfunction, the observed reductions in ALT and trends in AST, along with increased albumin concentrations, suggest potential hepatoprotective and protein-synthesis-supporting effects of the GB/MT supplementation, even under non-pathological conditions. These within-range alterations likely represent subtle physiological modulations rather than therapeutic effects, possibly attributable to the bioactive phytochemicals in GB/MT known to regulate hepatic enzymes and support liver function.

Regarding general health indicators (BUN, creatinine, triglycerides, and cholesterol), no statistically significant differences were found after supplementation with 1 or 2 g/d for 14 and 21 days or 7 days after withdrawal. These findings align with Gogulski et al. [22], who found that silybin extract supplementation did not alter BUN levels in dogs, and Gobalakrishnan et al. [32], who reported no changes in cholesterol levels in hypercholesterolemic rats administered 300 mg/kg of silybin. Additionally, Gogulski et al. [17] found that silybin supplementation had no effect on creatinine or cholesterol levels but did increase triglyceride values in dogs.

In our experiment, supplementation at 2 g/d for 14 and 21 days resulted in lower triglyceride and cholesterol values compared to baseline. The decrease in these values may be related to reduced production of these substances. However, the timing of sample collection may have influenced these results, as the animals were not fasted before blood samples were taken.

The experiment compared two levels of supplementation (1 and 2 g/d) of GB/MT in the diet and found that, prior to supplementation, dogs in the 2 g/d group had higher ALT values than those in the 1 g/d group. No significant differences were observed in other health indicators. Based on these results, using the 1 g/d extract is more cost-effective, as it did not result in significantly different ALT values compared to the 2 g/d extract. However, the results of this study obtained under controlled experimental conditions in a small cohort of healthy, neutered male and female dogs may not fully represent the broader clinical dog population encountered in veterinary practice.

The French Bulldogs enrolled in this study served as a representative model of healthy companion dogs. Therefore, the findings are primarily applicable to clinically healthy individuals. Nonetheless, based on the known hepatoprotective properties of GB and MT, these supplements may also be relevant to breeds predisposed to chronic hepatitis, such as Doberman Pinschers, English Springer Spaniels, English Cocker Spaniels, Labrador Retrievers, Dalmatians, American Cocker Spaniels, Cairn Terriers, Great Danes, and Samoyeds [33].

However, caution should be exercised when considering supplementation in dogs with genetic predispositions to specific health conditions. For example, GB has been associated with pro-convulsant activity in certain contexts and should be avoided in seizure-prone breeds such as Border Collies, Australian Shepherds, Belgian Tervurens, Labrador Retrievers, Golden Retrievers, Boxers, Beagles, German Shepherds, and Siberian Huskies [34]. Similarly, due to its potential antiplatelet effects, GB may not be appropriate for dogs predisposed to bleeding disorders, such as Doberman Pinschers, Scottish Terriers, Shetland Sheepdogs, German Shepherds, and Basset Hounds [35].

To date, literature regarding the optimal dosing strategies (continuous vs. cyclic supplementation) for these herbs in dogs remains limited. The doses used in this study were selected to fall well below levels reported to cause adverse effects. Importantly, no adverse effects were observed in any of the dogs throughout the study. All animals were monitored daily by a veterinarian to ensure their well-being.

It is also important to note that variability in measurement techniques between laboratories could affect the results, potentially leading to inconsistencies in findings across different studies. Furthermore, this study was conducted in healthy dogs, not those with induced liver disease, which may have limited the ability to clearly observe the effects of supplementation. In practice, diagnosing liver disease in dogs can be challenging, making it harder to detect the impact of supplementation. As a result, symptom-based treatment, including the use of appropriate feed and supplements, is commonly used in clinical practice when liver disease is suspected [17].

## 5. Conclusions

This study concluded that dietary supplementation of GB/MT seed extracts at 1 and 2 g/d for 14 and 21 days had no effect on antioxidant markers. However, it did affect liver function markers. Specifically, supplementation for 21 days led to a decrease in ALT values and an increase in serum albumin levels compared to pre-supplementation, while no significant changes were observed in other health markers. Furthermore, both supplementation levels (1 and 2 g/d) did not influence antioxidant markers, and the liver function effects were similar across both levels.

## Figures and Tables

**Table 1:** Effects of supplementation of GB and MT extract in diets on FI and BW in dogs.

Treatments	Feed Intake (% of Feed Given)				Initial Weight	Final Weight
	1–7 d	8–14 d	15–21 d	21–28 d	(kg)	(kg)
1 g/d	71.43 ± 12.37	78.57 ± 16.90	82.14 ± 11.74	85.72 ± 15.65	13.88 ± 1.84	14.78 ± 1.94
2 g/d	78.57 ± 7.14	73.81 ± 20.03	85.71 ± 7.83	83.33 ± 18.99	14.62 ± 1.95	14.50 ± 1.67
*p*-value	0.435	0.666	0.549	0.817	0.517	0.792

**Table 2:** Effects of supplementation of GB and MT extract at different periods in diets on the serum SOD levels of dogs (μ/mL).

Treatments	Periods				*p*-Value
	Day 0	Day 14	Day 21	Day 28	
1 g/day	10.19 ± 1.82	8.25 ± 3.97	10.47 ± 2.72	12.37 ± 2.61	0.201
2 g/day	11.18 ± 2.57	11.54 ± 2.33	11.52 ± 1.27	9.46 ± 3.82	0.393

**Table 3:** Effects of dietary supplementation of GB and MT extract at 1 g/day on liver function and health indicators in dogs.

Treatments	Liver Function					Health Indicators			
	ALT (IU/L)	AST (IU/L)	ALP (IU/L)	Total Prot (g/dL)	Albumin (g/dL)	BUN (mg/dL)	Creatinine (mg/dL)	Triglyceride (mg/dL)	Cholesterol (mg/dL)
Day 0	102.80 ± 16.08 ^a^	52.33 ± 19.78	26.00 ± 12.40	7.53 ±0.50	2.58 ± 0.50 ^b^	17.67 ± 1.97	1.73 ±0.79	81.75 ± 26.81	198.43 ± 89.48
Day 14	75.40 ± 25.12 ^ab^	52.50 ± 14.86	12.33 ± 6.15	7.83 ± 0.60	3.17 ± 0.26 ^ab^	22.17 ± 8.57	1.90 ± 1.02	92.95 ± 37.15	179.59 ± 24.47
Day 21	66.40 ± 11.87 ^b^	30.83 ± 5.42	17.00 ± 3.63	7.37 ± 0.98	3.28 ± 0.18 ^a^	19.17 ± 5.49	1.42 ± 0.78	75.04 ± 26.90	175.88 ± 21.13
Day 28	82.00 ± 10.89 ^ab^	69.17 ± 45.39	66.67 ± 50.07	7.77 ± 0.34	2.88 ± 0.59 ^ab^	19.83 ± 7.57	2.17 ± 0.55	87.24 ± 34.03	196.78 ± 38.99
Normal range	15–90	15–80	0–140	6.0–7.5	2.3–4.1	7.0–26.6	0.68–2.04	40–169	115–326
*p*-value	0.025	0.092	0.08	0.582	0.043	0.679	0.535	0.734	0.697

^a,b^ Means within the same column with different superscripts are significantly different (*p* < 0.05), alanine transaminase (ALT); aspartate aminotransferase (AST); alkaline phosphatase (ALP); total protein (Total prot); blood urea nitrogen (BUN).

**Table 4:** Effects of dietary supplementation of GB and MT extract at 2 g/day on liver function and health indicators in dogs.

Treatments	Liver Function					Health Indicators			
	ALT (IU/L)	AST (IU/L)	ALP (IU/L)	Total Prot (g/dL)	Albumin (g/dL)	BUN (mg/dL)	Creatinine (mg/dL)	Triglyceride (mg/dL)	Cholesterol (mg/dL)
Day 0	139.20 ± 29.24 ^a^	48.83 ± 19.63	19.33 ± 4.93	7.40 ± 0.20	2.60 ± 0.24 ^b^	21.00 ± 6.29	1.47 ± 0.40	112.23 ± 33.85	210.07 ± 72.02
Day 14	106.60 ± 49.57 ^ab^	47.17 ± 9.24	8.33 ± 4.73	7.48 ± 0.52	3.12 ± 0.45 ^ab^	20.17 ± 6.94	1.62 ± 0.91	70.30 ± 29.44	177.74 ± 33.27
Day 21	109.40 ± 41.31 ^ab^	36.67 ± 3.72	17.33 ± 3.21	7.80 ± 0.14	3.28 ± 0.37 ^a^	17.67 ± 6.19	1.60 ± 0.99	61.58 ± 19.28	175.57 ± 26.22
Day 28	69.40 ± 39.24 ^b^	51.50 ± 24.10	56.33 ± 60.93	7.60 ± 0.40	3.35 ± 0.29 ^a^	19.67 ± 5.32	2.62 ± 0.26	67.34 ± 51.41	146.39 ± 34.97
Normal range	15–90	15–80	0–140	6–7.5	2.3–4.1	7–26.6	0.68–2.04	40–169	115–326
*p*-value	0.010	0.218	0.323	0.284	0.000	0.804	0.084	0.17	0.243

^a,b^ Means within the same column with different superscripts are significantly different (*p* < 0.05), alanine transaminase (ALT); aspartate aminotransferase (AST); alkaline phosphatase (ALP); total protein (Total prot); blood urea nitrogen (BUN).

**Table 5:** Effects of supplementation of GB and MT extract in diets on liver function and health indicators in dogs.

Treatments	Liver Function					Health Indicators			
	ALT (IU/L)	AST (IU/L)	ALP (IU/L)	Total Prot (g/dL)	Albumin (g/dL)	BUN (mg/dL)	Creatinine (mg/dL)	Triglyceride (mg/dL)	Cholesterol (mg/dL)
Day 0 (before supplementation)									
1 g/d	98.33 ± 18.07 ^b^	52.33 ± 19.78	26.00 ± 12.41	7.53 ± 0.50	2.58 ± 0.50	22.17 ± 8.57	1.90 ± 1.02	92.95 ± 37.15	178.38 ± 22.09
2 g/d	139.20 ± 29.24 ^a^	48.83 ± 19.63	20.25 ± 4.43	7.53 ± 0.37	2.60 ± 0.24	20.17 ± 6.94	1.62 ± 0.91	79.16 ± 34.13	177.74 ± 33.27
*p*-value	0.019	0.765	0.407	1.000	0.942	0.666	0.623	0.519	0.969
Day 14									
1 g/d	75.40 ± 25.12	52.50 ± 14.86	12.33 ± 6.15	7.83 ± 0.60	3.17 ± 0.26	22.17 ± 8.57	1.90 ± 1.02	92.95 ± 37.15	178.38 ± 22.09
2 g/d	100.00 ± 47.19	47.17 ± 9.24	9.67 ± 4.32	7.48 ± 0.52	3.12 ± 0.45	20.17 ± 6.94	1.62 ± 0.91	79.16 ± 34.13	177.74 ± 33.27
*p*-value	0.324	0.472	0.405	0.329	0.819	0.666	0.623	0.519	0.969
Day 21									
1 g/d	66.40 ± 11.87	30.83 ± 5.42	17.00 ± 3.63	7.37 ± 0.98	3.28 ± 0.18	19.17 ± 5.49	1.42 ± 0.78	75.04 ± 26.90	177.54 ± 19.33
2 g/d	101.83 ± 41.34	36.67 ± 3.72	17.83 ± 3.31	7.73 ± 0.21	3.28 ± 0.37	17.67 ± 6.19	1.60 ± 0.99	67.16 ± 22.00	175.57 ± 26.22
*p*-value	0.092	0.055	0.687	0.392	1.00	0.666	0.728	0.591	0.886
Day 28 (7 days after supplementation)									
1 g/d	82.00 ± 10.89	69.17 ± 45.39	66.67 ± 50.07	7.77 ± 0.34	2.88 ± 0.59	19.83 ± 7.57	2.17 ± 0.55	87.24 ± 34.03	184.08 ± 46.74
2 g/d	66.00 ± 36.07	51.50 ± 24.10	37.60 ± 50.14	7.63 ± 0.37	3.35 ± 0.29	19.67 ± 5.32	2.62 ± 0.26	77.25 ± 51.99	146.39 ± 34.97
Normal range	15–90	15–80	0–140	6–7.5	2.3–4.1	7–26.6	0.68–2.04	40–169	115–326
*p*-value	0.367	0.419	0.363	0.531	0.113	0.966	0.110	0.702	0.145

^a,b^ Means within the same column with different superscripts are significantly different (*p* < 0.05), alanine transaminase (ALT); aspartate aminotransferase (AST); alkaline phosphatase (ALP); total protein (Total prot); blood urea nitrogen (BUN).

## Data Availability

Data are available from the corresponding author upon request.
